# Rapid Golgi Analysis Method for Efficient and Unbiased Classification of Dendritic Spines

**DOI:** 10.1371/journal.pone.0107591

**Published:** 2014-09-10

**Authors:** W. Christopher Risher, Tuna Ustunkaya, Jonnathan Singh Alvarado, Cagla Eroglu

**Affiliations:** 1 Department of Cell Biology, Duke University Medical Center, Durham, North Carolina, United States of America; 2 Department of Neurobiology, Duke University Medical Center, Durham, North Carolina, United States of America; 3 Duke Institute for Brain Sciences, Durham, North Carolina, United States of America; University of Nebraska Medical Center, United States of America

## Abstract

Dendritic spines are the primary recipients of excitatory synaptic input in the brain. Spine morphology provides important information on the functional state of ongoing synaptic transmission. One of the most commonly used methods to visualize spines is Golgi-Cox staining, which is appealing both due to ease of sample preparation and wide applicability to multiple species including humans. However, the classification of spines is a time-consuming and often expensive task that yields widely varying results between individuals. Here, we present a novel approach to this analysis technique that uses the unique geometry of different spine shapes to categorize spines on a purely objective basis. This rapid Golgi spine analysis method successfully conveyed the maturational shift in spine types during development in the mouse primary visual cortex. This approach, built upon freely available software, can be utilized by researchers studying a broad range of synaptic connectivity phenotypes in both development and disease.

## Introduction

Dendritic spines are the major sites for excitatory synaptic input in the central nervous system (CNS) [1]. During development, spines are known to undergo significant changes in morphology that are directly tied to changes in synaptic function [2]. Immature spine forms, including long, filopodia-type spines, are highly motile and are hypothesized to aid in the initiation of synaptic contact [3], [4]. By contrast, the fully mature mushroom spines are much more stable and contain an abundance of neurotransmitter receptors to sustain high levels of synaptic activity [2], [5]. Shifts in the progression of spine maturation can lead to alterations in neuronal circuit functioning, as reflected by the array of aberrant spine phenotypes observed in most neurological disorders [6], [7]. Thus the ability to effectively quantify and classify spines is of widespread interest for both developmental and translational neurobiology.

Several methods are currently used for the visualization and identification of dendritic spines, with one of the most prominent being Golgi-Cox staining [8]. Developed in 1873 by Camillo Golgi, this silver staining technique was used to great effect by Santiago Ramón y Cajal in his studies of nervous tissue ultrastructure [9]. Compared to newer techniques such as confocal microscopy of fluorescently-labeled neurons, the Golgi-Cox method maintains several advantages that give it broad appeal for the study of spine morphology. The staining can be applied to virtually any tissue, including postmortem human brain. Golgi-based image acquisition is available to any researcher with access to a simple light microscope and a camera, as opposed to the more expensive confocal microscopy setups. Additionally, Golgi-Cox images can be acquired much more rapidly than comparable confocal images, and Golgi-stained samples are viable for much longer periods of time (from months to years) than fluorescently-labeled tissue. However, despite these advantages there are still several major caveats that limit this method’s appeal. First, the sheer volume of quantifiable information from even small data sets makes image analysis extremely time-consuming. Second, consistency from analyst to analyst is low, owing to the subjectivity bias in categorizing spines with loosely-defined criteria. This could also affect data obtained by the same user, analyzing data over multiple days/months/years. Third, the commercial software currently available to identify and measure spines (e.g. Imaris, Neurolucida) is expensive, making the analysis cost-prohibitive for many researchers.

**Figure 1:**
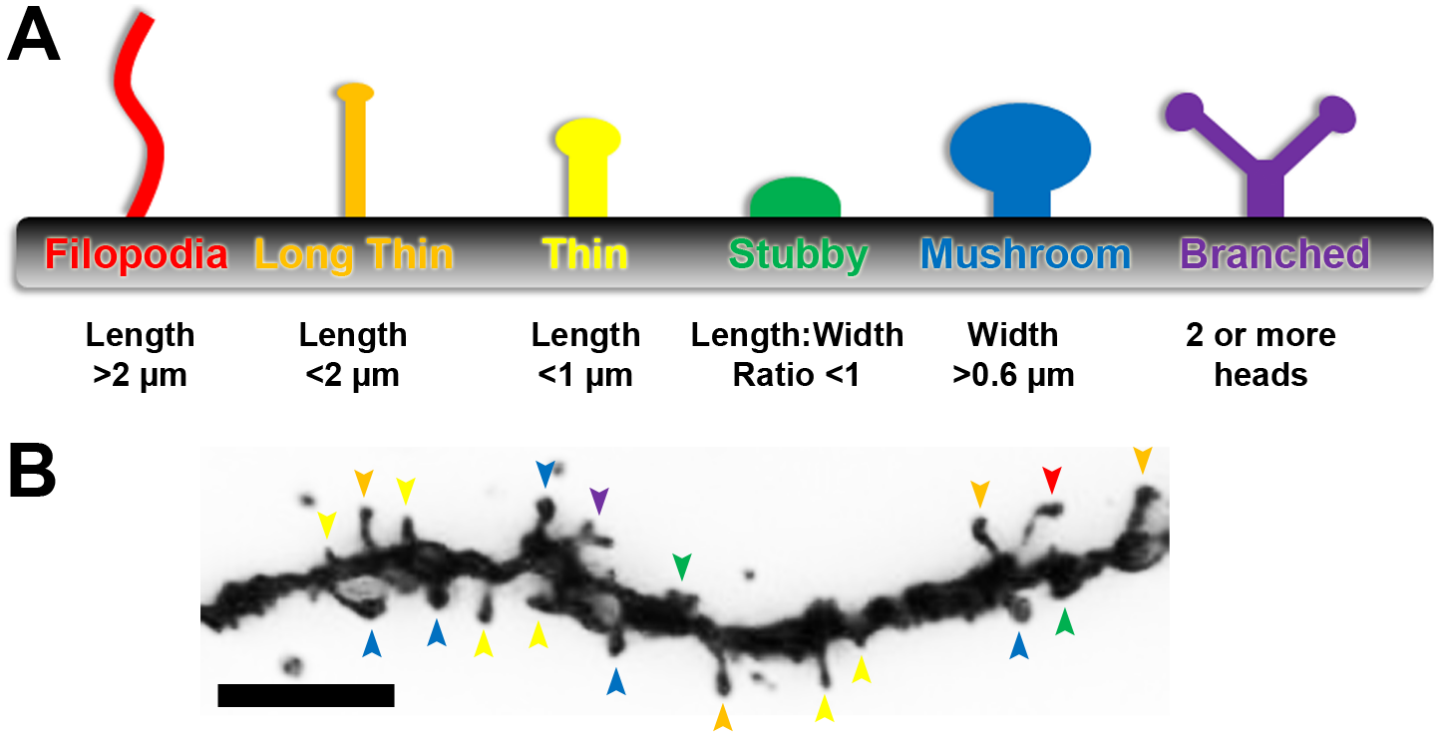
Geometric characteristics of dendritic spines allow for objective identification. (**A**) Common dendritic spines types found in the cortex. Spine maturity progresses (from left to right) from long, thin filopodia type structures (red) to wide-headed mushroom spines (blue) and the occasional branched spine (purple). Geometric characteristics of spines, listed below each type, are incorporated into the rapid spine analysis method. (**B**) Golgi-cox stained secondary dendritic branch of a Layer II/III pyramidal neuron in mouse primary visual cortex. Different spine types are indicated by arrowheads, color-coded to match **A**. Scale bar, 5 µm.

**Figure 2:**
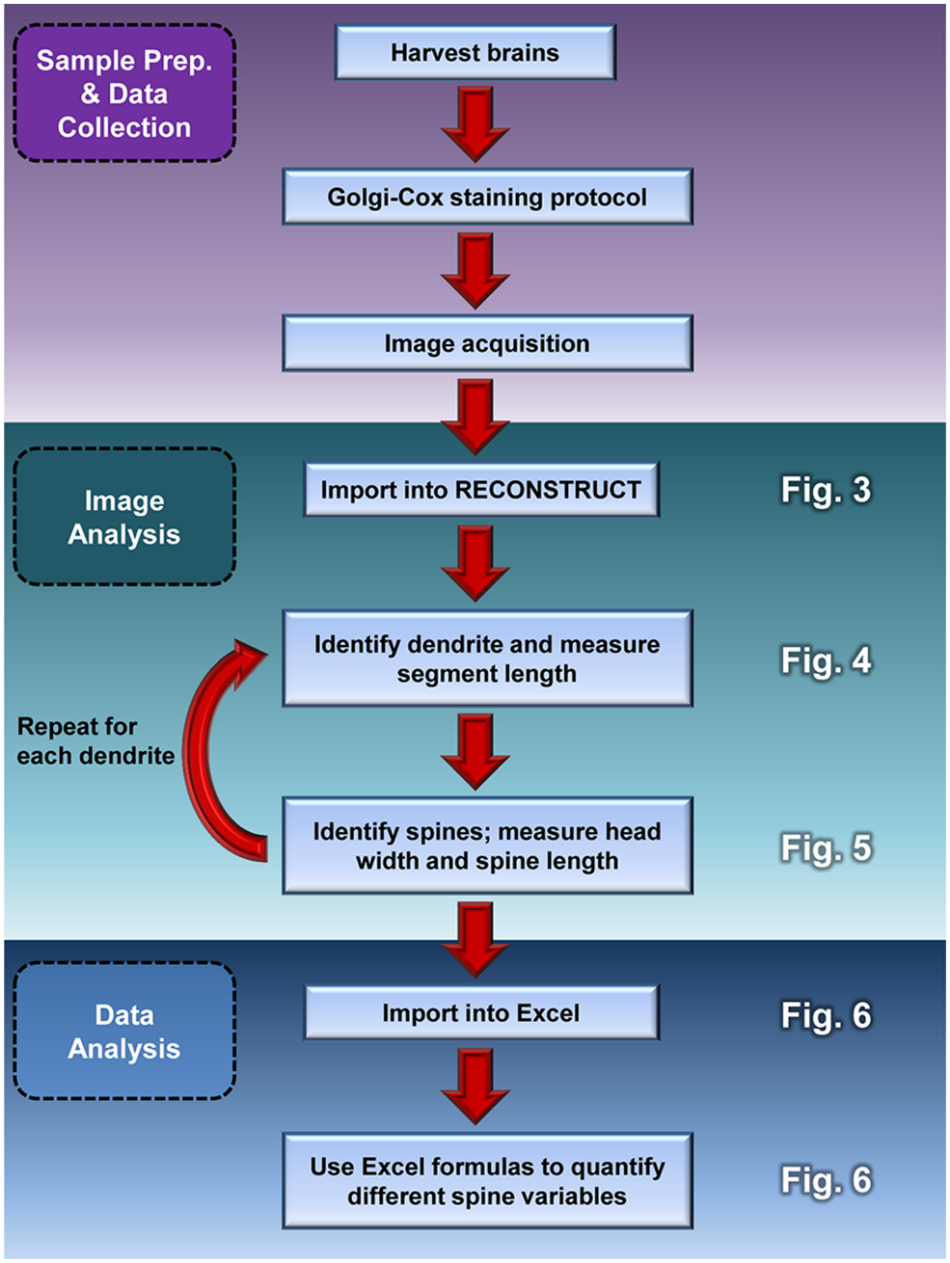
Flowchart for the rapid Golgi spine analysis method. Sample preparation and data collection (purple) are performed according to established protocols and manufacturer’s instructions. The rapid Golgi analysis method is split into two main parts. The first part, Image Analysis (teal), involves the importation of images into RECONSTRUCT and the measurement of dendrites and spines. These steps are repeated for each dendrite that is analyzed in a single Z-stack. The second part, Data Analysis (blue), uses custom formulas in Microsoft Excel to categorize spines and quantify numerous dendrite/spine variables. Figures corresponding to various sub-steps are listed on the right hand side of the chart.

Here, we present a method to circumvent these issues with the efficient and objective classification of dendritic spines from Golgi-Cox stained tissue. This approach, called the rapid Golgi spine analysis method, utilizes the distinct geometric characteristics of spines as the basis for their categorization (**Figure 1A**). By taking simple head width and neck length measurements, users can classify and quantify large numbers of spines in less time and with greater consistency than current methods (**Figure 1B**). Sample preparation and image acquisition are performed according to standard protocols, while the rapid Golgi spine analysis method employs freely available image analysis software and widely used spreadsheet software to quantify different spine variables with custom mathematical formulas (**Figure 2**). With this method, users can expect to spend considerably less time and conduct an unbiased classification of spines. Quantification achieved with this method can also be reliably compared to data obtained from other users, providing some much needed consistency to the analysis of spines.

## Methods

All experiments were conducted in accordance with the institutional animal care and use committee guidelines (IACUC Protocol Number A-185-08-11).

### Golgi-Cox staining

Brains were harvested whole from P14 or P25 mice on a 129 background and stained using the FD Rapid GolgiStain kit (FD NeuroTechnologies). Brains were rinsed with double distilled water and then immersed in a 1∶1 mixture of FD Solution A∶B for 2 weeks at room temperature in the dark. Brains were then transferred to FD Solution C and kept in the dark at 4°C for 48 hours. Solution C was replaced after the first 24 hours. In preparation for freezing, individual brains were placed in Peel-A-Way disposable embedding molds (VWR) and immersed in Tissue Freezing Medium (Triangle Biomedical Sciences). Dry ice was used to line the bottom of an ice bucket, which was then filled with 190-proof ethanol (Koptec). Using forceps, the molds were lowered into the ethanol (being careful not to allow the ethanol to spill into the top of the mold) and held until the TFM froze. Brains were kept at −80°C until sectioning. Cryosectioning was performed on a Leica CM 3050 S at −22°C. Coronal sections of 100 µm thickness were cut and transferred to gelatin coated slides (LabScientific) onto small drops of FD Solution C. This thickness enabled optimal staining and preservation of spines on the secondary and tertiary dendritic segments used for analysis in the examples presented here. However, any thickness between 80 to 240 µm (recommended in the FD Rapid GolgiStain instructions) can be used in order to satisfy the user’s unique requirements, providing that individual dendritic spines can still be differentiated and measured. After allowing sections to dry at room temperature in the dark for at least 4 hours (or overnight), slides were then stained exactly as described in the FD Rapid GolgiStain instructions (under “Part VI. Staining Procedure”). Permount (Fisher) was used for coverslipping.

### Imaging

Three independent coronal sections per each mouse, which contain the primary visual cortex (Bregma −2.5 to −3.2 mm, Interaural 1.3 to 0.6 mm [10]) were imaged. Layer II/III pyramidal neurons were identified by their distance from pia and their distinct morphologies. Secondary and tertiary dendrites of these neurons were selected for analysis. Z-stacks of Golgi-stained dendrites (up to 80 microns total on Z-axis; optical section thickness = 0.5 µm, i.e. 160 images per stack) were taken at 63x magnification on a Zeiss AxioImager M1. 5 Z-stacks were taken from each mouse.

### Rapid Golgi spine analysis

#### Step 1: Series import and calibration

**Figure 3:**
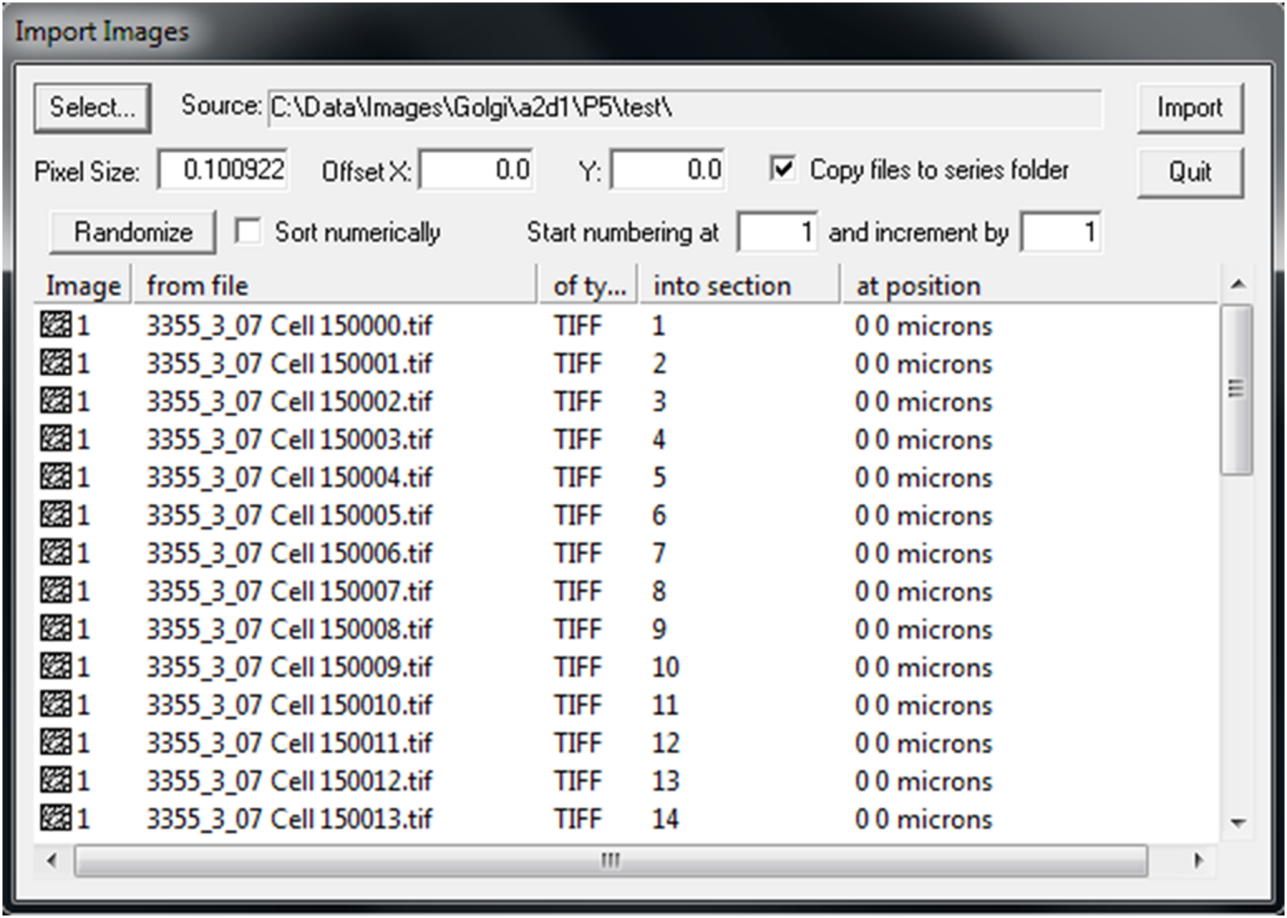
‘Import images’ dialog box in RECONSTRUCT. In this example, images from a Z-stack titled “3355_3_07 Cell 15″ are being loaded in sequential order. Pixel size (i.e. µm per pixel), once calibrated by the user, can be entered here or at any time after image importation.

In this step, you will import your Z-stack into an image analysis program for the rapid Golgi analysis method. Open a Z-stack in ImageJ (public domain software from the National Institutes of Health; http://imagej.nih.gov/ij/), convert it to RGB Color, and save it as an Image Sequence to a new folder. This image sequence will now be opened and analyzed in the freely available RECONSTRUCT software (http://synapses.clm.utexas.edu) [11]. After opening RECONSTRUCT, create a new “Series” for each Z-stack by going to ‘Series’->‘New’ and create a new.ser file, located in the same folder that contains the corresponding image sequence. Once the.ser file is created, import the Z-stack images by going to ‘Series’->‘Import’->‘Images’ (**Figure 3**), clicking the ‘Select’ button, selecting all of the files in the image sequence and clicking ‘Open’. From here, if pixel size (i.e. microns per pixel) is known, it can be entered into the ‘Pixel Size’ box; if not, this can be determined at a later point. Make sure Offset X and Y are each set to 0.0, and ‘Copy files to series folder’ is checked. Then click ‘Import’. The ‘Home’ button on the keyboard can be used to center and fit the current section on the screen, while the Page Up/Down buttons or mouse scroll wheel can be used to move up and down through the sections of the Z-stack. The ‘Pan and Zoom’ tool of RECONSTRUCT can be used to navigate within a single section. Details of other available commands can be found in the RECONSTRUCT help manual.

To set optical section thickness, go to ‘Section’->‘List sections’. This will show a list of all optical sections in the current z-stack. Highlight all of the sections, then go to ‘Modify’->‘Thickness’ and set the thickness in microns to 0.5. If Pixel Size was not set earlier, it can be performed now by drawing a line of known length and using the ‘Calibrate’ command (under ‘Trace’->‘Calibrate’). Enter the length of the line in microns, then choose the second option in the pop-up window (“Set pixel size to…”). Then enter the first and last section numbers to change pixel size for all sections in the Z-stack. Once this number is known, it can be entered during the image importing step for all future analyses (as long as the Z-stacks are imaged at the same magnification). The series is now calibrated and ready for analysis.

#### Step 2: Dendritic segment identification and measurement

**Figure 4:**
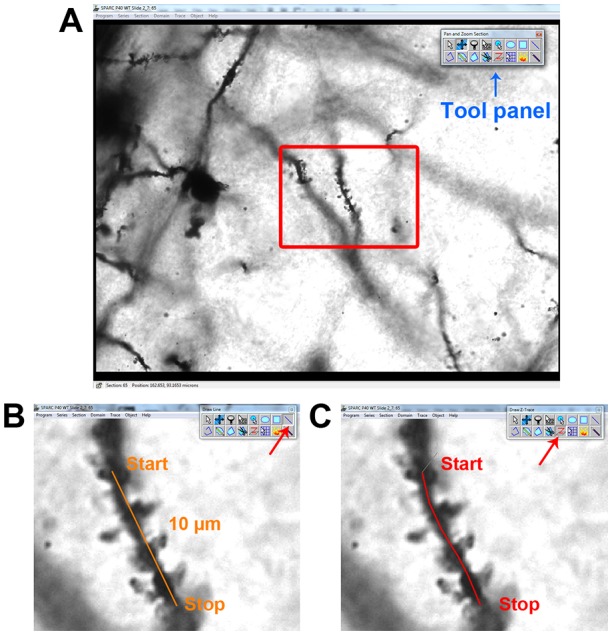
Dendrite identification and length measurements. (**A**) Main window in RECONSTRUCT. The tool panel can be seen in the upper right corner. The red rectangle indicates the dendritic segment chosen for analysis in this example. (**B**) Zoomed in image of the chosen dendritic segment. The ‘Draw Line’ tool has been selected to create the straight length measurement (∼10 µm) for this segment, with ‘start’ and ‘stop’ positions indicated. (**C**) The ‘Draw Z-Trace’ tool must be used to measure the Z-length of the dendritic segment. The ‘start’ and ‘stop’ positions created in **B** are used to guide the Z-trace.

Now you will identify and measure individual dendritic sections for analysis. Scroll up and down through the series to find dendritic sections (**Figure 4A**). An ideal section will be at least 10 microns in length, uninterrupted. Dendritic spines will be visually distinct from one another and have clearly defined spine heads (with the exception of head-less filopodia). Once a suitable stretch of dendrite has been chosen, open the Series Options (‘Series’->‘Options’) and click on the ‘Names/Colors’ tab. From here you can assign custom names to each of your lines (i.e. “traces”). To start, click on any of the buttons in the ‘Select or modify…’ section and assign it a specific name. In this example, we will start with a straight line measurement so give it a name like “d1_slen” (for dendrite 1, straight length). This line will not be used for any actual measurements but will serve as a reference for your ‘start’ and ‘stop’ positions when analyzing a specific dendritic segment. It will also establish which section will act as your ‘reference section’ when performing spine width measurements later on. Using the ‘Draw line’ tool, click at one end of the dendritic segment to be measured to establish your ‘start’ position (**Figure 4B**). Move your cursor approximately 10 microns along the length of the dendrite, using the live measurement at the bottom of the RECONSTRUCT window to determine the current length of the line. Then click a second time once you have reached 10 microns to close the trace at the ‘stop’ position (**Figure 4B**). You can double-check the length of this line by going to ‘Trace’->‘List traces’ and searching for the appropriately named trace. If the ‘Length’ column does not appear in the Trace list, go back to the Series Options and click on the ‘Lists’ tab, then make sure ‘Length’ is checked under the ‘Trace List’ section.

Because dendrites are rarely perfectly straight, you will want to draw a second line to more accurately trace the contours of the dendrite and obtain a more precise length measurement. Once again, return to the Series Options, choose a custom button and assign a name for this next measurement (e.g. “d1_zlen”, for dendrite 1, Z-trace length). This time you want to use the ‘Draw Z-Trace’ tool (**Figure 4C**). To draw the Z-trace, begin at the ‘start’ position that you established when drawing the straight line measurement previously. Scroll through the sections to find the section where the ‘start’ position is most clearly in focus, which is typically where the shaft appears the darkest. Click once to begin your Z-trace. Move the cursor down the middle of the dendrite towards your ‘stop’ position, clicking continuously while scrolling up/down through the series to keep the dendrite in focus. Once you have reached the ‘stop’ position (**Figure 4C**), click once more to extend the Z-trace to the ‘stop’ position, then right-click to end the Z-trace. You will not be able to see the Z-trace on the main RECONSTRUCT window at this stage. To visualize the Z-trace, go to ‘Object’->‘Z-traces’. Here you will see the length of all Z-traces in the Series (if the ‘Length’ column does not appear in the Z-Trace list, go back to the Series Options and click on the ‘Lists’ tab, then make sure ‘Length’ is checked under the ‘Z-Traces’ section). Double-click on a Z-trace name to open the ‘3D scene’ window. You will see a 3D representation of the Z-trace that can be manipulated with the mouse. Now that you have accurately measured the Z-length of your chosen dendritic segment, you can start measuring spines.

#### Step 3: Spine measurement

**Figure 5:**
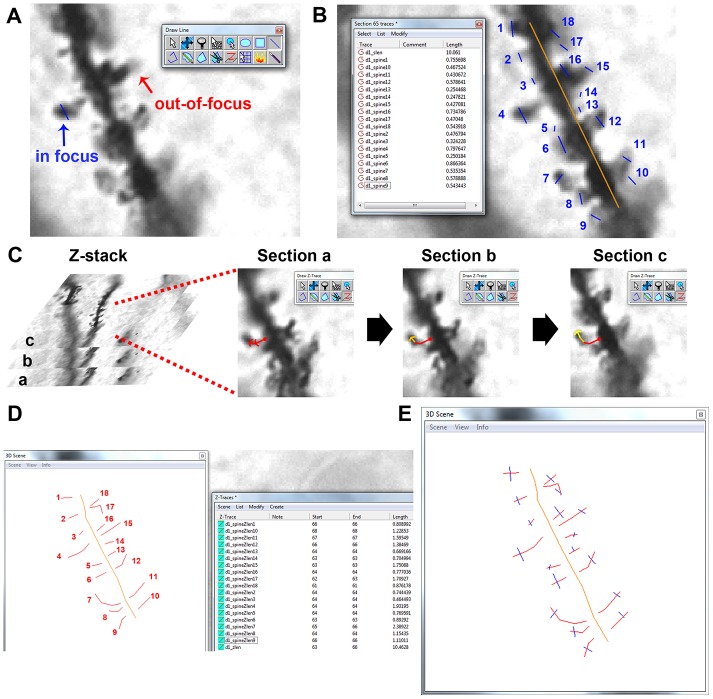
Measuring spines. (**A**) Due to the nonlinear nature of dendrites and spines, single optical sections will have a mixture of in focus (blue) and out-of-focus (red) spines. Spine width measurements, made by drawing a straight line across the widest part of the spine head, should only be drawn on sections where the spine is in focus. (**B**) Once all spines on the segment of interest have been found and measured, cutting and pasting all traces onto the same section as the reference line (orange) enables the creation of **Export List A** from the Trace List. Note that spines were marked in order going counter-clockwise around the reference line. (**C**) Drawing accurate Z-length measurements for spines often requires scrolling up and down through the Z-stack. In this example, the Z-trace starts at the base of the spine on Section a, continues down the neck of the spine through Section b, and terminates at the tip of the spine on Section c. (**D**) The Z-Trace List (used to create **Export List B**) yields the Z-length measurements for all analyzed dendrites and spines within the series. Z-traces can be visualized in the 3D Scene window by double-clicking on the name of the trace. Z-length measurements for this dendritic segment followed the same order for spines established in **B**. (**E**) Visualization of all Z-traces (red and orange) and straight line width traces (blue) for this segment.

This step outlines how to obtain width and length measurements of spines in RECONSTRUCT. You will be drawing straight lines to measure the head width of each spine on the segment; this line will also serve as a marker for each spine. Return to the Series Options and assign a new custom name, this time using the nomenclature “d*_spine+” (where “*” is the number of the dendritic segment that you are currently analyzing). The “+” in the trace name will force RECONSTRUCT to automatically increment numbers in consecutive traces, so the first trace will have a name like “d1_spine1”, the second trace will be named “d1_spine2”, and so on. Beginning at your ‘start’ position, locate each dendritic spine and scroll through the series to find the section where the spine head is most in focus (**Figure 5A**). On this section only, use the ‘Draw line’ tool to draw a straight line across the width of the spine head, perpendicular to the long axis of the spine (**Figure 5A**). Then move on to the next spine and repeat this measurement process. Move systematically along the length of the dendrite towards the ‘stop’ position, staying on one side of your straight reference line (**Figure 5B**). Once you reach the ‘stop’ position, switch to the spines on the opposite side of the reference line and make your way back to the ‘start’ position. Maintaining this order will be essential for matching these width measurements with the spine length measurements that you will take next. At any point, you can bring up the Trace List (**Figure 5B**) to see what spines have been measured and what their widths are, keeping in mind that spines may be on different sections. If any of the spines were branched, i.e. multiple spine heads attached to a single spine neck (**Figure 1A**), you must note this in the Trace List. To do this, click on the trace name of the branched spine, go to ‘Modify’->‘Attributes’, and add “branch” to the end of the trace name (e.g. “d1_spine1branch”). This re-naming will be important when classifying spines in a later step. Once you have drawn width measurements for all spines along the segment, you need to transfer the width traces to the same optical section (the one with the reference line). To do this, open the Trace List, scroll through the series and select all width traces that are not on the reference section, then cut (Ctrl+X) and paste (Ctrl+V) them to the reference section (**Figure 5B**). You may have to repeat this several times to move all traces to the reference section. Once all traces have been moved to the reference section, go to ‘List’->‘Save’ in the Trace List window and save the current trace list as a Comma Separated Value (.csv) file. Give it a name that identifies it with a specific dendritic segment (e.g. “d1_traces.csv”). This file, which contains the spine head width values for an individual dendrite, will hereafter be referred to as **Export List A**.

Now that you have completed the spine width measurements, spine length measurements are next. Start by opening the Series Options and assigning a name with the nomenclature “d*spineZlen+” (where “*” is the number of the dendritic segment that you are currently analyzing). You will be using the ‘Draw Z-Trace’ tool to obtain a 3D measurement for spine length. For each spine, you will want to follow a similar approach to when you drew the Z-trace for the dendritic segment itself (**Figure 4C**). Begin by scrolling through the Z-stack (**Figure 5C**) to find the optical section where the base of the spine (i.e. where the spine neck connects to the dendritic shaft) is most in focus. Click at the base of the spine to initiate the Z-trace, then move the cursor down the middle of the spine towards the tip (**Figure 5C**). Click continuously while scrolling up/down through the series to keep the spine in focus. Once you have reached the tip of the spine, click once more to extend the Z-trace to the edge of the spine tip, then right-click to end the Z-trace (**Figure 5C**). In order to match the length measurements with the width measurements taken earlier, make sure to measure spines in the same order as previously established (**Figure 5D, E**). Spine length measurements can be viewed in the Z-Trace list (**Figure 5D**). If you are going to analyze more dendrites in the Series, start again from Step 2, creating a new straight reference line (**Figure 4B**) with a new dendrite name. Once you have finished analyzing all desired dendrites in the Series, export the length measurements by opening the Z-Trace List, selecting ‘List’->‘Save’, and saving the Z-Trace list as a.csv file (e.g. “ztraces.csv”). This list, hereafter referred to as **Export List B**, contains Z-length information for all dendrites analyzed throughout the Series.

#### Step 4: Constructing the data set

**Figure 6:**
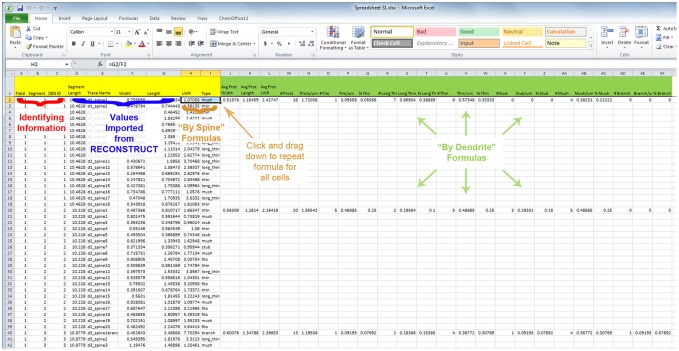
Categorizing spines and characterizing dendrites in Excel. The provided spreadsheet template (**Spreadsheet S1**) contains all of the formulas required to utilize the measurements obtained from RECONSTRUCT. Identifying information (red) allows the user to specify each analyzed dendrite according to their own conventions. The ‘DEN ID’ column must be unique to each dendrite in a data set for the proper working of the included formulas. Values imported from RECONSTRUCT (blue) are obtained from Export Lists A & B. “By Spine” formulas (gold), including ‘LWR’, or length-to-width ratio, and ‘Type’, which classifies spines according to a custom hierarchical formula, should be dragged down and repeated for each row (i.e. spine) of the data set. “By Dendrite” formulas (green), which measure average protrusion width, length, LWR, and protrusion density, should be copied and pasted only onto the first line of each new DEN ID value.

With image analysis complete, you can proceed with data analysis of the obtained measurements. The 2D traces (e.g. widths) and Z-traces (e.g. lengths) should now be imported into Microsoft Excel. For convenience, a preconfigured spreadsheet (**Spreadsheet S1**; **Figure 6**) is available to organize your data. In this spreadsheet, information is presented with a single spine on each row, along with the associated measurements and identifying information. In the example shown in **Figure 6**, a single Z-stack (“Field 1″) contains 3 dendritic segments (1–3) that were analyzed. Dendritic segment 1 has 17 spines whose names appear under the “Trace Name” column. The “DEN ID” column is required to assign a unique identification value to each individual dendrite in a data set. Having a unique DEN ID for each dendritic segment in a data set is necessary for the formulas that will be used to calculate protrusion density and other values. Segment lengths (**Figure 4C**) can be obtained from Export List B for the related Z-stack. Trace names for each spine should be copied and pasted from the parent dendrite’s Export List A, along with the associated spine head width value from the same file. The “Length” column should be populated with the associated values from Export List B. Make sure that all 3 columns, “Trace Name”, “Width”, and “Length”, are matched up for each analyzed spine (this will be easy if you followed the same order of analysis for both width and length measurements during Step 3). Continue importing values from Export Lists A and B until all analyzed dendrites have been input.

Once all information has been copied into the spreadsheet, it is now a simple matter of copying formulas to complete the analysis. “LWR”, or length-to-width ratio, is simply the length value divided by the width value. “Type” is a critical formula for determining spine type based on width, length, and LWR values of an individual spine. The syntax for the formula is as follows:
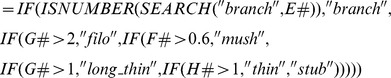
where # is the row number, E is the Trace Name, F is the spine width value, G is the spine length value, and H is the LWR. The formula is hierarchical, classifying spines in the following order: 1) “branch” for branched spine, when “branch” appears in the trace name as entered manually by the user; 2) “filo” for filopodia, when the length value >2 µm; 3) “mush” for mushroom spine, when the width value >0.6 µm; 4) “long_thin” for long thin spine, when the length value >1 µm; 5) “thin” for thin spine, when the LWR value >1; 6) “stub” for stubby spine, when the LWR value ≤1. The “LWR” and “Type” formulas, known as the “By Spine” formulas, can be dragged down to each cell in the data set (**Figure 6**). The remaining formulas, e.g. protrusions per micron, average protrusion width and length, etc., are the “By Dendrite” formulas and should be copied and pasted onto the first line of each unique DEN ID (**Figure 6**). These formulas use the DEN ID value for each dendritic segment as a basis for their calculations. Once the “By Dendrite” formulas have been copied and pasted for each DEN ID in the data set, the results can be exported to your preferred statistics program for further analysis.

### Tutorial Data Set

The example dendrite used in this article is available as part of a “Tutorial Set” that can be used to familiarize the user with this method. Three dendritic segments have been analyzed, and their measurements have been exported to.csv files as described above. This “Tutorial Set”, available as a. zip file containing all relevant images, series files and trace files, can be freely downloaded at http://tinyurl.com/kxxg3qh.

### Statistics

Statistical comparisons were made using Student’s t-test in Statistica (Statsoft).

## Results and Discussion

**Figure 7:**
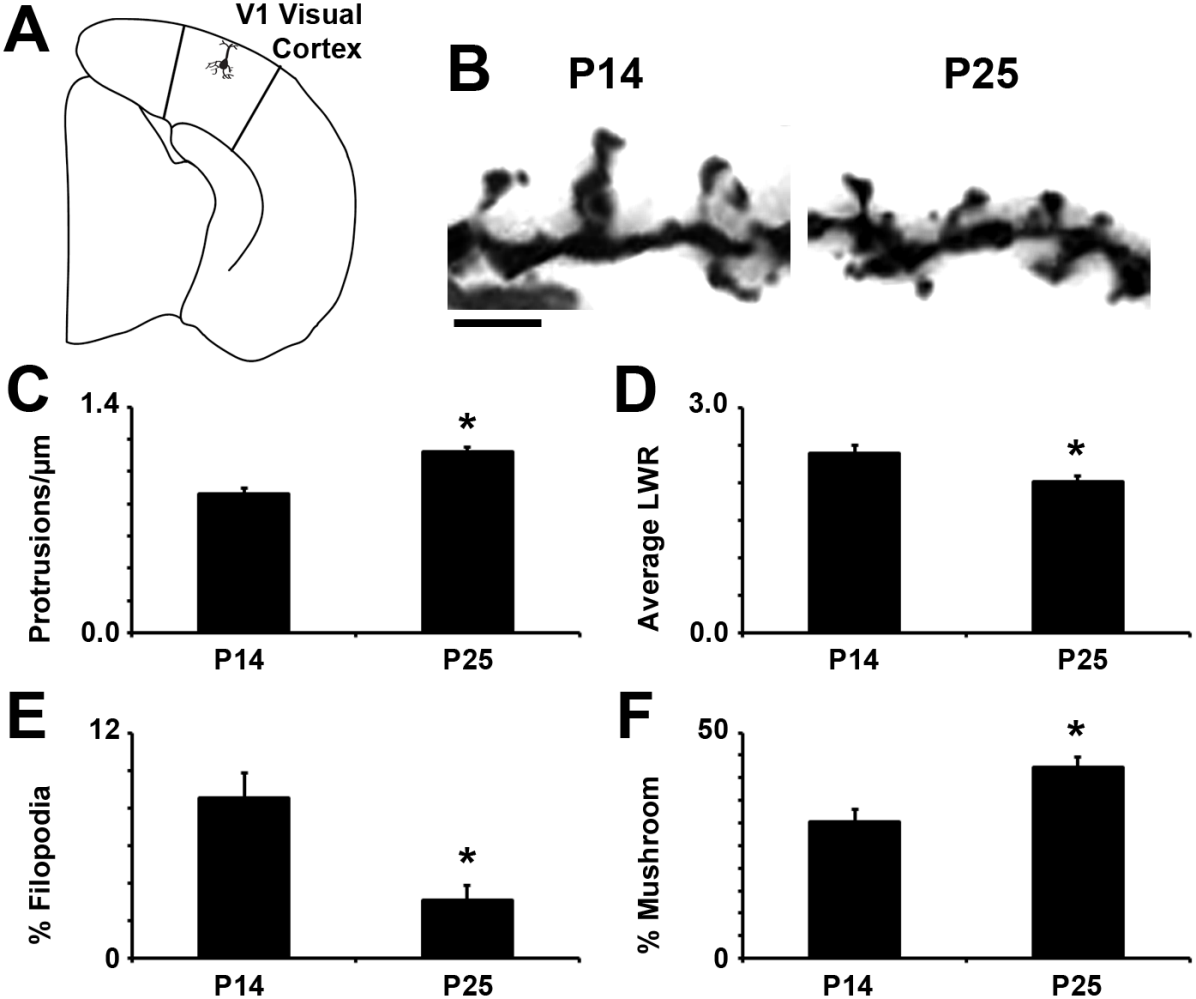
The rapid Golgi spine analysis method accurately reports spine proliferation and maturation. (**A**) Diagram of the right hemisphere of the mouse brain, sectioned coronally, showing the location of V1. Secondary and tertiary dendrites of Layer II/III pyramidal neurons were analyzed via the rapid spine analysis method. (**B**) Representative images of Golgi-Cox stained dendritic spines at P14 and P25. Spines at P25 appear shorter and more abundant than their P14 counterparts. Scale bar, 2 µm. (**C**) Protrusion density significantly increases between P14 and P25 (P<0.05) while (**D**) average LWR decreases, reflecting shorter, wider spines (P<0.05). (**E**) The percentage of immature filopodia-type spines sharply decreases between P14 and P25 (P<0.05), offset by (**F**) an increase in the proportion of mature mushroom spines (P<0.05).

The rapid Golgi spine analysis method provides a subjective and efficient way to classify large numbers of spines in a data set. To validate this method, we analyzed spines on secondary and tertiary dendrites from Layer II/III pyramidal neurons in mouse primary visual cortex (V1; **Figure 7A**). Cortical neurons are well-accepted to increase in spine density over the first several postnatal weeks [12]. In addition, there is a shift in the shape of spines with development, typified by a shortening of the neck and widening of the head (**Figure 7B**). In comparing spines from P14 to P25 cortical neurons, we found that the rapid spine analysis method confirmed both an increase in protrusion density with developmental age (**Figure 7C**) and an overall change in the average shape of spines, represented as a decrease in the length-to-width ratio (**Figure 7D**). Taken further, the formulas developed as part of the rapid spine analysis method were also able to confirm maturation-associated changes in spine types. Long, thin filopodia-type spines significantly decreased from P14 to P25 (**Figure 7E**). By contrast, there was a significant increase in the percentage of mature, mushroom-type spines by P25 (**Figure 7F**). Taken together, these changes are in line with what one would expect from a neuronal network undergoing significant refinement and maturation.

As an extra validation for the spine classification formula developed for this method, we compared the results from P14 pyramidal neurons obtained from the formula against spine classifications previously made by eye (i.e. classifying individual spines as filopodia, mushroom, thin, etc. in the absence of actual measurements) from the same dendrites. In this comparison, there was no difference in filopodia (0.07±0.01 filopodia/µm by formula versus 0.06±0.01 by eye; p = 0.50) or mushroom density (0.25±0.02 mushrooms/µm by formula versus 0.28±0.02 by eye; p = 0.37), showing that the formula accurately replicated spine classifications made by a trained observer (W.C.R.).

Though it can be a powerful tool for the study of neuronal ultrastructure, Golgi-Cox staining has a number of drawbacks. Perhaps the biggest hindrance to a wider acceptance of this method is the time commitment involved. The staining process itself can take up to 2.5 weeks, followed by the time required to image the Golgi-stained slides. Though the rapid spine analysis method will not expedite these first crucial steps, it will streamline the analysis steps to follow. Once the images have been obtained, it can be a daunting task to sort through dozens of Z-stacks and find dendritic segments for analysis. By focusing on the simple acquisition of width and length measurements, the rapid spine analysis method takes the guesswork out of spine classification. This allows for a much more efficient throughput of large data sets. Another significant drawback to Golgi-Cox staining is the inherent differences in spine classification from analyst to analyst. In our experience, individuals can assign highly differing spine classifications even when analyzing the same dendrites. This subjectivity makes it difficult if not impossible to compare results from different analysts, which can be a significant concern for experiments with extremely large data sets that are to be analyzed by more than one individual. Even commercial programs such as Neurolucida (MBF Bioscience) are dependent on this highly subjective method of spine classification. With the rapid spine analysis method, spine classification is performed with an algorithm that takes into account only the length and width measurements of the analyzed spines, significantly diminishing categorical subjectivity and user-to-user variability. Another significant advantage of the rapid spine analysis method is more accurate Z-length measurements, since you can trace the entire contour of the spine in three-dimensions rather than just draw straight lines from the base to the tip. Finally, rapid Golgi spine analysis uses the freely-available software RECONSTRUCT and the widely used Microsoft Excel spreadsheet program, making the Golgi technique more widely accessible to scientists since it does not rely on expensive commercial software.

In summary, the rapid Golgi spine analysis method takes an unbiased, measurement-based approach to the typically subjective technique of dendritic spine classification. With this method, large data sets can be reliably and efficiently analyzed by multiple users without fear of classification bias inherent between individuals. This approach cannot replace higher resolution techniques for studying neuronal morphology such as green fluorescent protein (GFP) transgenic mice or intracellular injections of fluorescent markers. Nevertheless, Golgi-Cox staining is commonly used to study dendritic spines in all types of brain tissue, and our method provides a reproducible, quantitative means to perform this analysis. Deficits in spine density and morphology have been described in the brains of patients with neurological disorders [13] as well as in transgenic animal knockouts of synapse-associated proteins [7], [14], [15]. Thus the method presented here should have broad application for neuroscientists studying synaptic connectivity in both development and disease.

## Supporting Information

Spreadsheet S1 **This spreadsheet contains all of the required formulas that are needed to run the rapid Golgi spine analysis technique.** As shown in **Figure 6**, identifying information (red) allows the user to specify each analyzed dendrite according to their own conventions. The ‘DEN ID’ column must be unique to each dendrite in a data set for the proper working of the included formulas. Values imported from RECONSTRUCT (blue) are obtained from Export Lists A & B. “By Spine” formulas (gold), including ‘LWR’, or length-to-width ratio, and ‘Type’, which classifies spines according to a custom hierarchical formula, should be dragged down and repeated for each row (i.e. spine) of the data set. “By Dendrite” formulas (green), which measure average protrusion width, length, LWR, and protrusion density, should be copied and pasted only onto the first line of each new DEN ID value. The spreadsheet is filled with measurements from the supplied “Tutorial Set” to familiarize new users with the process of completing their own data set.
(XLSX)

## References

[1] Harris KM, Kater SB (1993) Dendritic spines: cellular specializations imparting both stability and flexibility to synaptic function. Ann Rev Neurosci 17: 341–371. 10.1146/annurev.ne.17.030194.002013 8210179

[2] Nimchinsky EA, Sabatini BL, Svoboda K (2001) Structure and function of dendritic spines. Ann Rev Physiol 64: 313–353. 10.1146/annurev.physiol.64.081501.160008 11826272

[3] Dunaevsky A, Tashiro A, Majewska A, Mason C, Yuste R (1999) Developmental regulation of spine motility in the mammalian central nervous system. Proc Natl Acad Sci U S A 96: 13438–13443. 10557339 10.1073/pnas.96.23.13438 PMC23966

[4] Ziv NE, Smith SJ (1996) Evidence for a role of dendritic filopodia in synaptogenesis and spine formation. Neuron 17: 91–102. 8755481 10.1016/s0896-6273(00)80283-4

[5] Matsuzaki M, Ellis-Davies GC, Nemoto T, Miyashita Y, Iino M, et al (2001) Dendritic spine geometry is critical for AMPA receptor expression in hippocampal CA1 pyramidal neurons. Nat Neurosci 4: 1086–1092. 11687814 10.1038/nn736 PMC4229049

[6] Fiala JC, Spacek J, Harris KM (2002) Dendritic spine pathology: cause or consequence of neurological disorders? Brain Res Rev 39: 29–54. 12086707 10.1016/s0165-0173(02)00158-3

[7] Cruz-Martín A, Crespo M, Portera-Cailliau C (2010) Delayed stabilization of dendritic spines in fragile X mice. J Neurosci 30: 7793–7803. 20534828 10.1523/JNEUROSCI.0577-10.2010 PMC2903441

[8] Das G, Reuhl K, Zhou R (2012) The Golgi-Cox method. 1018: 313–321. 10.1007/978-1-62703-444-9_29 23681640

[9] Ramon Y Cajal S (1951) Structure and connections of neurons. Bull Los Angeles Neurol Soc 17: 5–46. 14944970

[10] Franklin KBJ, Paxinos G (2001) The mouse brain in stereotaxic coordinates. New York: Academic Press.

[11] Fiala JC (2005) Reconstruct: a free editor for serial section microscopy. J Microsc 218: 52–61. 15817063 10.1111/j.1365-2818.2005.01466.x

[12] Holtmaat AJ, Trachtenberg JT, Wilbrecht L, Shepherd GM, Zhang X, et al (2005) Transient and persistent dendritic spines in the neocortex in vivo. Neuron 45: 279–291. 15664179 10.1016/j.neuron.2005.01.003

[13] Irwin SA, Patel B, Idupulapati M, Harris JB, Crisostomo RA, et al (2001) Abnormal dendritic spine characteristics in the temporal and visual cortices of patients with fragile-X syndrome: a quantitative examination. Am J Med Genet 98: 161–167. 11223852 10.1002/1096-8628(20010115)98:2<161::aid-ajmg1025>3.0.co;2-b

[14] Kwon H-BB, Kozorovitskiy Y, Oh W-JJ, Peixoto RT, Akhtar N, et al (2012) Neuroligin-1-dependent competition regulates cortical synaptogenesis and synapse number. Nat Neurosci 15: 1667–1674. 23143522 10.1038/nn.3256 PMC3536444

[15] Ma Y, Ramachandran A, Ford N, Parada I, Prince DA (2013) Remodeling of dendrites and spines in the C1q knockout model of genetic epilepsy. Epilepsia 54: 1232–1239. 23621154 10.1111/epi.12195 PMC3700654

